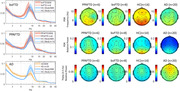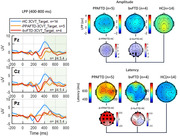# Comparative EEG biosignatures in Alzheimer’s disease and frontotemporal dementia: A pilot study

**DOI:** 10.1002/alz.090543

**Published:** 2025-01-09

**Authors:** Amir H. Meghdadi, Ajay Verma, Mario F. Mendez, Chris Berka

**Affiliations:** ^1^ Advanced Brain Monitoring, Carlsbad, CA USA; ^2^ Formation Venture Engineering, Boston, MA USA; ^3^ UCLA David Geffen School of Medicine, Los Angeles, CA USA; ^4^ Advanced Brain Monitoring, Inc., Carlsbad, CA USA

## Abstract

**Background:**

Alzheimer's disease (AD) and frontotemporal dementia (FTD) are common causes of dementia, but differentiating between them can be challenging due to overlapping symptoms [1]. Quantitative electroencephalography (EEG) is emerging as a promising tool to identify potential biosignatures that can distinguish AD and FTD [2]–[5]. Prior EEG research has revealed slowing of the posterior dominant rhythm (PDR) in both AD and FTD patients compared to controls, reflecting underlying neurodegeneration. Our study aimed to compare quantitative EEG measures during resting‐state and attention tasks between bvFTD, PPAFTD, AD patients, and controls to determine if distinctive EEG/ERP profiles exist that can aid differential diagnosis.

**Methods:**

Participants included individuals with behavioral‐variant Fronto‐temporal dementia (bvFTD, n=8, ages 42‐78), Primary Progressive Aphasia Frontotemporal dementia (PPAFTD, n=6, ages 53‐75), Alzheimer’s dementia (AD, n=20, ages 58‐79), and controls with normal cognition (HC, n=14, ages 47‐78). Twenty‐channel resting‐state EEG with eyes‐closed was collected during 5‐minutes of wakefulness (STAT X24). A subset of participants also completed a 3‐Choice‐vigilance‐task (3CVT) ERP attention task. Power spectral densities (PSD) during resting‐state and ERP waveforms elicited by the ERP task were computed and compared between groups.

**Results:**

bvFTD on average exhibited a slowing of the posterior‐dominant rhythm (PDR) similar to the slowing observed in the AD group. However, unlike the AD group that showed a significant reduction in the power of the PDR (alpha power), both FTD groups had alpha power similar to HC. In the ERP attention task, FTD groups exhibited a delayed and reduced late positive potential (LPP) compared to controls.

**Conclusions:**

We identified distinctive EEG biosignatures associated with Frontotemporal Dementia (FTD) and Alzheimer's Disease (AD). Quantitative analysis of the posterior dominant rhythm (PDR) revealed that both AD and FTD patients exhibited slowing of the PDR frequency. However, the power of PDR was significantly reduced in the AD while relatively preserved in FTD. FTD subtypes may also demonstrate distinct EEG abnormalities such as more pronounced PDR slowing in bvFTD/ more delayed event‐related potential (ERP) components in PPAFTD. Additional research is still needed to validate the reliability and specificity of these EEG biosignatures in differentiating FTD subtypes from each other and from AD.